# The Utility of Platelet Indices in Predicting Multiorgan Dysfunction in Scrub Typhus

**DOI:** 10.1155/2020/3870354

**Published:** 2020-07-31

**Authors:** Saravanakumari Vijayakumar, Stalin Viswanathan, Dheeraj Jain

**Affiliations:** ^1^Department of Pathology, Sri Lakshmi Narayana Institute of Medical Sciences and Hospital, Kudapakkam Post, Pondicherry 605502, India; ^2^Department of General Medicine, JIPMER, Pondicherry 605009, India; ^3^Department of Medicine, Indira Gandhi Medical College and Research Institute, Kathirkamam, Pondicherry 605006, India

## Abstract

Platelet indices have been used to diagnose and prognosticate infections such as *tuberculosis*, malaria, dengue, and septic shock. Platelet indices have previously not been used in the prediction of multiorgan dysfunction (MODS) in patients with scrub typhus. A three-year retrospective review of patient charts was performed. Patients with and without MODS were compared. Platelet indices and other clinical and laboratory variables were used in logistic regression analysis to determine significant predictors. A ROC curve was generated with the platelet indices to predict MODS. Of 189 patients, 106 were male. Respiratory rate, serum creatinine, liver function tests, platelet count, thrombocytopenia <150 × 109/L, mean platelet volume (MPV) > 7.3 fL, and plateletcrit ≤0.19% varied significantly between patients with MODS and those without. Platelet indices are inexpensive and easily available. Only thrombocytopenia along with creatinine, alanine transaminase, and abnormal chest radiograph could significantly predict MODS in patients with scrub typhus.

## 1. Introduction

Platelets are fragments of megakaryocytes, 1500–2000 of them being formed from a single megakaryocyte. Its physiological roles include hemostasis, inflammation, wound healing, and angiogenesis [1]. Hundreds of proteins and peptides are secreted from the three types of granules within the platelets. Platelet indices are biomarkers of platelet activation and they include mean platelet volume (MPV), platelet distribution width (PDW), plateletcrit (PCT), mean platelet component, mean platelet mass, platelet component distribution width, platelet larger cell ratio, and immature platelet fraction. The geometric mean of the log transformation of the platelet volume distribution curve gives the MPV in femtolitres; it can also be obtained from the ratio of the plateletcrit to platelet count [1]. A cut-off at 20% of the platelet size distribution width gives the PDW. The volume occupied by platelets in the blood, calculated as a percentage, is the PCT. Multiorgan dysfunction syndrome(MODS) is defined by the presence of dysfunction of two or more organ systems, and in scrub typhus, the prevalence has varied among studies (1/5^th^ to 1/3^rd^ of patients) [2–4]. MODS in scrub typhus has been compared with non-MODS groups only in one adult and pediatric study each, and platelet indices were not used in either study [2, 4]. Platelet indices have been used in the diagnosis and prognosis of various inflammatory, infective, and malignant disorders [1]. Infections that have been studied include septic shock [5], urinary tract infection [6], *tuberculosis* [7], *H. pylori* [8], spontaneous bacterial peritonitis [9], surgical site infections [10], hepatitis [11], pneumonia [12], malaria [13], and dengue [14]. Platelet indices have not been previously studied in patients with scrub typhus, and hence we hypothesized whether these indices could predict multiorgan dysfunction syndrome in scrub typhus.

## 2. Materials and Methods

This retrospective cross-sectional study was conducted at Indira Gandhi Medical College and Research Institute, a government-funded teaching hospital in Pondicherry. The study aimed to find out whether platelet indices were useful in predicting multiorgan dysfunction (MODS) in patients with scrub typhus. Ethical approval was obtained prior to commencement of the study. The study period was between 01 January 2015 to 31 December 2018. All patients who had presented with an acute febrile illness of less than three weeks' duration and had a confirmed diagnosis of scrub typhus based on a Scrub IgM ELISA (InBios) OD of >0.500 with or without eschar and recovered with either doxycycline or azithromycin therapy were recruited for the study. A sample for complete blood count (CBC) was obtained in all patients at the time of admission along with other investigations such as urea, creatinine, liver function tests, electrocardiogram, and chest radiograph. The CBC was performed using a Celltac Alpha MEK-6400 automated hematology analyser within two hours of obtaining a 2 mL venepuncture sample into an EDTA tube.

### 2.1. Definitions

A systolic pressure of <90 mmHg was taken as shock. A platelet count of <150 × 10^9^/L at admission was considered as thrombocytopenia. Cut-offs for plateletcrit, PDW, and MPV were taken as ≤0.19, >16.3, and >7.3 respectively, using median values from a study based on South Indian blood donors [15]. A new increase in creatinine of ≥0.3 mg/dL compared to baseline was considered as acute kidney injury (AKI), according to AKIN criteria [16]. Elevation of transaminases (both aspartate transaminase [AST] and alanine transaminase [ALT] ≥ 200 U/L) was considered as hepatic dysfunction. New-onset pulmonary infiltrates, pleural effusion, or consolidation was considered as radiographic signs of scrub typhus. A pO_2_/FiO_2_ of ≤300 with radiographic infiltrates < one week was considered as acute lung injury/acute respiratory distress syndrome according to Berlin criteria [17]. Neck stiffness with or without altered sensorium/cranial nerve palsy and cerebrospinal fluid (CSF) cell count of lymphocytes >10/hpf and elevated protein >60 mg/dL were taken as meningitis. Multiorgan dysfunction was considered when there were two or more organs involved. The qSOFA score was calculated based on the presence of one or more of the following: hypotension <100 mmHg, respiratory rate ≥22/minute, and altered sensorium (GCS <15).

### 2.2. Statistics

Demographic and clinical data, hematological, renal, and hepatic parameters, arterial blood gases, radiograph, and electrocardiographic (ECG) findings were noted in an Excel spreadsheet. IBM SPSS for Windows v22 was used for statistical analysis. Frequencies of organ dysfunction (clinical and laboratory) were calculated and chi-square analysis was used to compare categorical variables. Means ± SD were calculated, and Student's *t*-test was used to compare continuous variables of patients with multiorgan dysfunction and those without. Logistic regression analysis was performed to determine whether the platelet indices along with other clinical and laboratory parameters were able to predict MODS. A ROC was plotted with the platelet indices individually as the test variable and MODS as the state variable to calculate AUC. For the association of the clinical and laboratory variables in relation to the qSOFA scores, a one-way ANOVA and chi-square analysis were performed for continuous and categorical variables, respectively. A *P*-value of ≤0.05 was considered statistically significant.

## 3. Results

There were 189 patients in the study, with males constituting 56.1% (*n* = 106). One hundred and fifty patients had neurological symptoms (headache, neck pain, seizures, weakness, or altered sensorium), while 106 had musculoskeletal symptoms (myalgia, arthralgia, or arthritis). Comparatively, lesser numbers were seen with symptoms of other systems: gastrointestinal (*n* = 95; vomiting, abdominal pain, bleeding, or jaundice), cardiorespiratory (*n* = 91; dyspnea, orthopnea, cough, hemoptysis, or pleurisy), and genitourinary (*n* = 21; oligoanuria or hematuria). The commonest clinical finding was of hepatomegaly (*n* = 60), followed by eschar in 59 patients. Thirty-one had regular alcohol use, while eight had diabetes mellitus. Among the various indicators and contributors of organ dysfunction, thrombocytopenia (*n* = 108), pneumonitis on radiography (*n* = 35), AKI (*n* = 19), hepatitis dysfunction (*n* = 27), meningitis (*n* = 13), shock (*n* = 9), and ARDS (*n* = 3) were observed in decreasing order of frequency. Three patients died, two with MODS and another with intracranial bleed with an associated alcohol withdrawal syndrome (without MODS), who presented with fever and intolerable headache.

**Figure 1:**
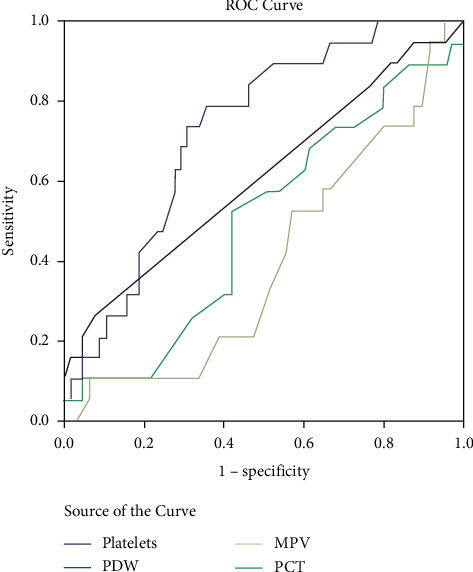
ROC curve for platelet indices vs. MODS. PDW: platelet distribution width; MPV: mean platelet volume; PCT: plateletcrit.

Transaminases, alkaline phosphatase, albumin, creatinine, platelet counts, MPV >7.3, and plateletcrit ≤0.19 differed significantly in patients with MODS compared to those without (Table 1). Respiratory rate, crackles, hepatomegaly, creatinine, AST, ALT, alkaline phosphatase, albumin, hemoglobin, thrombocytopenia (150 × 10^9^/L), plateletcrit ≤0.19, and MPV >7.9, and abnormal chest radiograph were able to significantly predict MODS on univariate logistic regression analysis (Table 2). On multivariate analysis, only thrombocytopenia, creatinine, ALT, and abnormal radiographs were able to predict so (Table 3). When the ROC was plotted for the platelet indices against MODS, only platelet count with a cut-off of 124 × 10^9^/L had a significant area under the curve (AUC) with ability to predict MODS with a sensitivity and specificity of 73.7% and 70%, respectively (Table 4 and Figure 1).

## 4. Discussion

Platelet indices are routinely measured in automated hematology analysers at no extra costs and have been studied in a wide array of settings involving inflammatory diseases, infections, and malignancies [18]. The most commonly used such marker is MPV; large platelets have more granules and adhesion molecules and are associated with more adverse events [19]. MPV has been studied in various viral, bacterial, mycobacterial, and fungal infections [18]. Our patients with and without MODS did not vary significantly with respect to MPV, but an MPV cut-off of >7.3 fL was significant for MODS on regression analysis. This was a lower cut-off compared to that of other studies. In a study by Gao et al., an MPV >10.5 was a predictor of mortality in septic shock [5]. MPV had high sensitivity and specificity for predicting spontaneous bacterial peritonitis with a cut-off of 8.7 at 95.9% and 91.7%, respectively, in cirrhotics [9]. The MPV correlated with C-reactive protein (CRP) levels in patients with pneumonia as well as *tuberculosis* [7, 12]. In a study by Camara-Lemarroy et al., MPV had 82% sensitivity and 78% and specificity to differentiate bacterial meningitis from tuberculous meningitis [20]. MPV as a utility in dengue has varied with different studies [14]. In Brazil, a higher MPV was found with the first vivax malaria infection and those with symptoms for >3 days [20].

Thrombocytopenia is a common manifestation in tropical infections such as dengue, malaria, leptospirosis, and scrub typhus. Rapidly dropping platelet counts in dengue and thrombocytopenia <50 × 10^9^/L in malaria have been considered as indicators of severe disease [21, 22]. In a North Indian study on scrub typhus, thrombocytopenia was the commonest component of those patients fulfilling criteria of severe sepsis [23]. The same study found that hemoglobin and total leukocyte counts were the only hematological parameters useful in predicting organ failure. A similar finding with anemia and leucocytosis predicting severe scrub typhus was seen from South Korea [24]. In our study, thrombocytopenia significantly predicted MODS in both univariate and multivariate regression analysis and with moderate sensitivity using the ROC. Similar to Ritin et al., thrombocytopenia was also the biggest component of MODS (as hematological dysfunction) in our study [23]. MODS was seen in 57 of our patients; if thrombocytopenia was excluded from the MODS criteria, the number of patients with MODS came down to 28.

Platelet distribution width indicates platelet volume variability [25]. Activated platelets with pseudopodia increase PDW. PDW has been used in the identification of pulmonary *tuberculosis* and acute cholecystitis and organ failure in acute pancreatitis [25]. Increased PDW was seen in ascitic fluid infection, vivax malaria, dengue, and septic shock [5, 9, 13, 14]. PDW was reduced in deep-seated surgical site infection in orthopaedic patients and children with Gram-positive organism-related urinary tract infection [6, 10]. In our study, there was no difference in PDW among patients with MODS and those without.

Plateletcrit is the least studied among the four platelet parameters. They have been found to be decreased in dengue and septic shock [5, 14]. A plateletcrit cut-off of ≤0.19 was significant between patients with MODS and those without.

There are a few studies wherein severe scrub typhus has been studied with regard to predictors of severity and mortality [24, 26–28]. However, platelet indices have not been used previously to predict MODS in scrub typhus. Our patients were of a similar age to that present in the Chennai study, [28] but younger than that of Korean patients [24, 26]. We had a larger patient cohort comparable to Kim et al. (189 vs. 208). Serum creatinine was a predictor in our study as in Kim et al. [24]. Age and absence of an eschar were risk factors for severe disease in studies by Premraj et al. and Kim et al., while these factors were not predictive in our study [24, 28]. TNF-*α* was used to predict the severity of scrub typhus by Lee Kim et al. [26] APACHE II scores were used by Lee Kim et al. and Griffith et al., which we could not perform due to arterial blood gases (ABG) data being unavailable in many patients. Respiratory complications were commoner in the study by Griffith et al., where patients requiring intensive care were studied. We had 15 admissions into ICU without the need for ventilation or renal replacement therapy, and the findings were comparable to Kim et al. There was no mortality in the study by Premraj et al., while we had three deaths (1.5%) in comparison to other studies: 0.004% in Kim et al., 24.9% in Griffith et al., and 18.5% in Lee Kim et al. The patients in our study had qSOFA scores of 0, 1, or 2. There was none with a score of 3. The scores correlated well with systolic blood pressure, respiratory rate, leukocyte counts, and hepatic dysfunction achieving statistical significance, but not with mortality or ICU admission (Tables 1 and 5). Though there was no association of the qSOFA scores with any of the platelet indices, the median score in patients with MODS was higher than in those without MODS, reflecting its use in scrub typhus-related multiorgan failure as well.

Regression analysis was used to determine whether platelet indices could predict individual organ dysfunction. A PCT ≤0.19 predicted both respiratory (*P*=0.05) and hepatic dysfunction (*P*=0.04), while PDW predicted hepatic dysfunction (*P*=0.01) alone.

### 4.1. Limitations

Since the study was retrospective, the dataset of ABG was not available for all patients. Lack of ABG precluded us from calculating APACHE scores, which would have given us a better measure of clinical severity in these patients. Only qSOFA scores could be calculated based on the available data. The MPV may be affected by preanalytical variables and this could not be nullified in a retrospective study. Standardized values of the platelet indices for the South Indian population are not available. Cut-offs for PCT, MPV, and PDW were taken from the median values based on a study of blood donors in the South Indian population [15]. Higher cut-offs for these indices have been used in other studies. No large population-based studies are available for reference. Also, the reference values may vary with the analyser being used. We do not have follow-up data on any patient's repeat IgM titers, since the test may become positive in patients from endemic areas with recent scrub typhus, and the diagnosis was based on a single IgM ELISA done at admission.

## 5. Conclusions

A simple, noninvasive, and inexpensive test is often required when one needs to prognosticate common infections that are potentially fatal, such as scrub typhus, more so when the limitations are due to the lack of diagnostic facilities for elaborate tests. We attempted to study platelet indices as an inexpensive test for this purpose. The qSOFA scores did not have any significant association with any of the platelet indices, but rather with the leukocyte count and LFT. Among the platelet-related indices, only platelet counts were useful in prognosticating scrub typhus-associated MODS. Thrombocytopenia in conjunction with ALT, creatinine, and abnormal CXR predicted MODS in patients with scrub typhus.

## Figures and Tables

**Table 1:** Clinical and laboratory parameters and organ dysfunction.

Variables	With MODS (*n* = 57)	Without MODS (*n* = 132)	Significance
Males (*n*)	32	74	0.99
Age (years)	40.70 ± 14.48	37.89 ± 15.15	0.23
Duration of stay (days)	5.21 ± 2.46	4.75 ± 1.67	0.21
Duration of symptoms (days)	9.26 ± 3.75	8.96 ± 4.12	0.63
Temperature (C)	38.29 ± 0.94	38.55 ± 0.95	0.08
Pulse rate (beats/min)	101 ± 16.7	91.19 ± 13.2	0.42
Systolic blood pressure (mm·Hg)	113.61 ± 27.82	108.05 ± 14.14	0.15
Respiratory rate (breaths/min)	20.25 ± 15.19	15.17 ± 9.8	0.03
Creatinine (mg/dL)	0.90 ± 0.52	0.66 ± 0.28	0.002
Albumin (g/dL)	2.92 ± 0.95	3.53 ± 0.71	<0.001
Aspartate aminotransferase (U/L)	124.23 ± 95.36	77.08 ± 64.83	0.002
Alanine aminotransferase (U/L)	120.02 ± 81.86	73.08 ± 59.43	0.003
Alkaline phosphatase (U/L)	163.81 ± 108.46	116.30 ± 64.15	0.006
Hemoglobin (g/dL)	11.43 ± 2.16	12.21 ± 2.50	0.03
White blood cell count (×10^9^/L)	9.79 ± 6.21	9.06 ± 4.75	0.38
Platelets (×10^9^/L)	118.32 ± 68.97	165.32 ± 87.55	<0.001
Thrombocytopenia (*n*)	49	59	<0.001
Platelet distribution width (fL)	17.17 ± 1.87	17.35 ± 1.02	0.58
Platelet distribution width >16.3 (*n*)	55	127	0.92
Mean platelet volume (fL)	6.42 ± 1.15	6.02 ± 1.25	0.26
Mean platelet volume >7.39 fL (*n*)	43	74	0.01
Plateletcrit (%)	0.09 ± 0.03	0.11 ± 0.02	0.10
Plateletcrit ≤0.19% (*n*)	18	63	0.04
Electrocardiographic heart rate (beats/min)	95.14 ± 21.60	91.48 ± 14.09	0.39
Meningitis (*n*)	10	3	<0.001
Acute respiratory distress syndrome (*n*)	2	1	0.16
qSOFA scores (mean ± SD)	0.65 ± 0.64	0.39 ± 0.54	0.01
Score 0 (*n*)	84	25	0.02
Score 1 (*n*)	44	27	
Score 2 (*n*)	4	5	
Acute kidney injury (*n*)	15	4	<0.001
Shock (*n*)	6	3	0.014
Pneumonitis (*n*)	32	3	<0.001
Hepatitis (*n*)	12	10	0.08
Mortality (*n*)	2	1	0.16

**Table 2:** Logistic regression analysis to predict MODS in scrub typhus.

Variables	Significance	Odds ratio (95 CI)
Platelet distribution width	0.53	0.862 (0.541–1.374)
Mean platelet volume	0.79	1.066 (0.665–1.706)
Plateletcrit	0.57	0.024 (0.913–1.308)
Plateletcrit ≤0.19%	0.04	1.978 (1.028–3.807)
Platelet distribution width ≥16.3	0.92	0.924 (0.174–4.907)
Thrombocytopenia <150 × 10^9^/L	<0.001	0.132 (0.058–0.300)
Mean platelet volume >7.3 fL	0.01	0.415 (0.207–0.832)
Respiratory rate	0.01	1.038 (1.007–1.071)
Crackles	<0.001	12.7 (4.446–36.280)
Hepatomegaly	0.04	0.516 (0.269–0.988)
Serum creatinine	<0.001	4.753 (2.027–11.147)
Aspartate aminotransferase	0.001	1.007 (1.003–1.012)
Alanine aminotransferase	<0.001	1.009 (1.004–1.015)
Alkaline phosphatase	0.003	1.007 (1.002–1.012)
Serum albumin	<0.001	0.428 (0.271–0.678)
Hemoglobin	0.04	0.875 (0.767–0.997)
Abnormal chest radiograph	<0.001	0.031 (0.011–0.087)

**Table 3:** Multivariate regression analysis to predict MODS in scrub typhus.

Variables	Significance	Odds ratio (95% CI)
Thrombocytopenia	0.002	17.66 (2.648–117.812)
Creatinine	<0.001	9.43 (3.054–29.166)
Alanine aminotransferase	0.006	1.027 (1.008–1.046)
Abnormal chest radiograph	<0.001	29.72 (10.071–87.720)

**Table 4:** Area under the curve for platelet indices with respect to scrub typhus.

Variables	Area under the curve	Significance
Platelets	0.727	0.003 (0.610–0.843)
Platelet distribution width	0.499	0.991 (0.354–0.645)
Mean platelet volume	0.401	0.190 (0.261–0.541)
Plateletcrit	0.611	0.143 (0.458–0.764)

**Table 5:** qSOFA scores in relation to clinical and laboratory variables.

Variables	0 (*n* = 109)	1 (*n* = 71)	2 (*n* = 9)	Significance
Age (median)	37	34	21	0.71
Duration of symptoms (days)	8.7 ± 4.0	9.7 ± 3.9	7.8 ± 3.8	0.16
Duration of stay (days)	4.8 ± 2.0	4.8 ± 1.3	6.2 ± 3.4	0.11
Pulse rate (beats/min)	92.4 ± 14.9	93.7 ± 13.5	95.1 ± 14.4	0.77
Systolic blood pressure (mmHg)	110.2 ± 14.1	111.8 ± 24.7	86.6 ± 11.1	0.001
Respiratory rate (breaths/min)	11.0 ± 7.7	22.7 ± 13.3	24.8 ± 3.8	<0.001
Creatinine (mg/dL)	0.7 ± 0.3	0.7 ± 0.4	0.6 ± 0.3	0.52
Aspartate aminotransferase (IU/L)	77.4 ± 66.2	96.8 ± 79.3	88.4 ± 102.0	<0.001
Alanine aminotransferase (IU/L)	76.3 ± 60.3	91.3 ± 74.7	157.0 ± 86.6	0.003
Alkaline phosphatase (IU/L)	124.3 ± 93.1	130.4 ± 63.0	181.1 ± 48.0	0.13
Serum protein (g/dL)	6.8 ± 0.8	6.8 ± 0.9	6.2 ± 0.5	0.16
Hemoglobin (g/dL)	12.7 ± 2.4	12.0 ± 2.2	10.6 ± 2.8	0.21
WBC ×10^9^/L	8.672 ± 5.346	10.484 ± 5.088	7.055 ± 3.141	0.03
Platelets ×10^9^/L	144.820 ± 81.294	161.600 ± 91.269	141.500 ± 52.915	0.41
PDW	17.2 ± 1.3	17.3 ± 1.0	18.2 ± 0.5	0.60
MPV (fL)	5.9 ± 1.1	6.5 ± 1.4	5.5 ± 0.3	0.12
PCT (%)	0.10 ± 0.10	0.10 ± 0.13	0.11 ± 0.02	0.65
ICU admission (*n*)	10	4	1	0.64
Meningitis (*n*)	8	6	1	0.90
Acute respiratory distress syndrome (*n*)	3	0	0	0.32
Acute kidney injury (*n*)	10	9	0	0.44
Shock (*n*)	0	5	4	<0.001
Hepatitis (*n*)	13	8	1	0.99
Mortality (*n*)	2	1	0	0.94

## Data Availability

The data used to support the findings of this study are openly available in Figshare at http://doi.org/10.6084/m9.figshare.11353985.

## References

[1] Budak Y. U., Polat M., Huysal K. (2016). The use of platelet indices, plateletcrit, mean platelet volume and platelet distribution width in emergency non-traumatic abdominal surgery: a systematic review. *Biochemia Medica*.

[2] Zhao D., Zhang Y., Yin Z., Zhao J., Yang D., Zhou Q. (2017). Clinical predictors of multiple organ dysfunction syndromes in pediatric patients with scrub typhus. *Journal of Tropical Pediatrics*.

[3] Vivekanandan M., Mani A., Priya Y. S., Singh A. P., Jayakumar S., Purty S. (2010). Outbreak of scrub typhus in Pondicherry. *The Journal of the Association of Physicians of India*.

[4] Varghese G. M., Trowbridge P., Janardhanan J. (2014). Clinical profile and improving mortality trend of scrub typhus in South India. *International Journal of Infectious Diseases*.

[5] Gao Y., Li Y., Yu X. (2014). The impact of various platelet indices as prognostic markers of septic shock. *PLoS One*.

[6] Akya A., Rostami-Far Z., Chegene Lorestani R. (2019). Platelet indices as useful indicators of urinary tract infection. *Iranian Journal of Pediatric Hematology & Oncology*.

[7] Lee M. Y., Kim Y. J., Lee H. J., Cho S. Y., Park T. S. (2016). Mean platelet volume inMycobacterium tuberculosisInfection. *BioMed Research International*.

[8] Baxendell K., Walelign S., Tesfaye M. (2019). Association between infection with *Helicobacter pylori* and platelet indices among school-aged children in central Ethiopia: a cross-sectional study. *BMJ Open*.

[9] Abdel-Razik A., Eldars W., Rizk E. (2014). Platelet indices and inflammatory markers as diagnostic predictors for ascitic fluid infection. *European Journal of Gastroenterology & Hepatology*.

[10] Zhang Z., Ji Y., Wang Z., Qiu X., Chen Y. (2018). The association between platelet indices and deep surgical site infection after open induction internal fixation for traumatic limb fractures. *Infection and Drug Resistance*.

[11] Pan Y., Muheremu A., Wu X., Liu J. (2016). Relationship between platelet parameters and hepatic pathology in patients with chronic hepatitis B infection-a retrospective cohort study of 677 patients. *Journal of International Medical Research*.

[12] Lee J.-H., Yoon S. Y., Kim H.-S., Lim C. S. (2015). Characteristics of the mean platelet volume, neutrophil to lymphocyte ratio, and C-reactive protein compared to the procalcitonin level in pneumonia patients. *Platelets*.

[13] Leal-Santos F. A., Silva S. B., Crepaldi N. P. (2013). Altered platelet indices as potential markers of severe and complicated malaria caused by Plasmodium vivax: a cross-sectional descriptive study. *Malaria Journal*.

[14] Mukker P., Kiran S. (2018). Platelet indices evaluation in patients with dengue fever. *International Journal of Research in Medical Sciences*.

[15] Naina H. V. K., Harris S. (2006). Platelet and red blood cell indices in harris platelet syndrome. *Blood*.

[16] Lopes J. A., Jorge S. (2013). The RIFLE and AKIN classifications for acute kidney injury: a critical and comprehensive review. *Clinical Kidney Journal*.

[17] Umbrello M., Formenti P., Bolgiaghi L., Chiumello D. (2017). Current concepts of ARDS: a narrative review. *International Journal of Molecular Sciences*.

[18] Viswanathan S., Saravanakumari V. (2016). Are platelet indices useful in diagnosis of tropical acute febrile illnesses?. *Journal of Local and Global Health Science*.

[19] Korniluk A., Koper-Lenkiewicz O. M., Kamińska J., Kemona H., Dymicka-Piekarska V. (2019). Mean platelet volume (MPV): new perspectives for an old marker in the course and prognosis of inflammatory conditions. *Mediators of Inflammation*.

[20] Cámara-Lemarroy C. R., Delgado-Garcia G., De La Cruz-Gonzalez J. G., Villareal-Velazquez H. J., Gongora-Rivera F. (2017). Mean platelet volume in the differential diagnosis of tuberculous and bacterial meningitis. *The Journal of Infection in Developing Countries*.

[21] White N. J., Elizabeth A., Jameson L., Kasper D., Longo D., Fauci A., Hauser S., Loscalzo J. (2018). *Malaria*.

[22] WHO (2012). *Handbook for Clinical Management of Dengue*.

[23] Sharma R., Mahajan S. K., Singh B., Raina R., Kanga A. (2019). Predictors of severity in scrub typhus. *The Journal of the Association of Physicians of India*.

[24] Kim D.-M., Kim S. W., Choi S.-H., Yun N. R. (2010). Clinical and laboratory findings associated with severe scrub typhus. *BMC Infectious Diseases*.

[25] Wang F., Meng Z., Li S., Zhang Y., Wu H. (2017). Platelet distribution width levels can Be a predictor in the diagnosis of persistent organ failure in acute pancreatitis. *Gastroenterology Research and Practice*.

[26] Kim H. L., Park H. R., Kim C.-M., Cha Y. J., Yun N. R., Kim D.-M. (2019). Indicators of severe prognosis of scrub typhus: prognostic factors of scrub typhus severity. *BMC Infectious Diseases*.

[27] Griffith M., Peter J. V., Karthik G. (2014). Profile of organ dysfunction and predictors of mortality in severe scrub typhus infection requiring intensive care admission. *Indian Journal of Critical Care Medicine: Peer-Reviewed, Official Publication of Indian Society of Critical Care Medicine*.

[28] Premraj S., Mayilananthi K., Krishnan D., Padmanabhan K., Rajasekaran D. (2018). Clinical profile and risk factors associated with severe scrub typhus infection among non-ICU patients in semi-urban south India. *Journal of Vector Borne Diseases*.

